# Neurotoxic effects of different sizes of plastics (nano, micro, and macro) on juvenile common carp (*Cyprinus carpio*)

**DOI:** 10.3389/fnmol.2022.1028364

**Published:** 2022-10-20

**Authors:** Mohamed Hamed, Christopher J. Martyniuk, Mervat Naguib, Jae-Seong Lee, Alaa El-Din H. Sayed

**Affiliations:** ^1^Department of Zoology, Faculty of Science, Al-Azhar University (Assiut Branch), Asyut, Egypt; ^2^Interdisciplinary Program in Biomedical Sciences Neuroscience, Department of Physiological Sciences, Center for Environmental and Human Toxicology, College of Veterinary Medicine, UF Genetics Institute, University of Florida, Gainesville, FL, United States; ^3^Department of Zoology, Faculty of Science, Assiut University, Asyut, Egypt; ^4^Department of Biological Sciences, College of Science, Sungkyunkwan University, Suwon, South Korea

**Keywords:** nanoplastics, neurotoxicity, histopathology, acetyl cholinesterase, nitric oxide, common carp

## Abstract

Using common carp as a model, we assessed the effects of polyethylene (PE) plastics on the brain. We measured activity of acetylcholinesterase (AChE), monoamine oxidase (MAO), and the content of nitric oxide (NO) in carp brain following exposure to 100 mg/L of either macroplastics (MaP), microplastics (MPs), or nanoplastic (NPs) for 15 days compared to an unexposed group. Following exposure, each biochemical biomarker was reduced 30–40%, with a higher magnitude of change corresponding to the smaller size of the particles (NPs > MPs > MaPs). In the carp tectum, exposure for 15 days to plastic particles caused varying degrees of necrosis, fibrosis, changes in blood capillaries, tissue detachment, edema, degenerated connective tissues, and necrosis in large cerebellar neurons and ganglion cells. In the carp retina, there was evidence for necrosis, degeneration, vacuolation, and curvature in the inner layer. Here we provide evidence that exposure to plastic particles can be associated with neurotoxicity in common carp.

## Introduction

Global plastic production over the past several decades has increased significantly (Plastics Europe, 2011, 2015) and an estimated 10% of plastics produced annually are deposited into rivers, seas, and oceans (Barnes et al., 2009). Nanoplastics in these aquatic systems is estimated to comprise 60–80% of the total marine debris (Derraik, 2002). Plastics enter water systems via waste management, sewage treatment plants, and aerial deposition (Nowack and Bucheli, 2007). Plastic debris affects more than six hundred (660) species due to ingestion and entanglement; thus, it is a significant aquatic pollutant (GEF, 2011). Plastic material in aquatic ecosystems degrades into smaller and smaller pieces via physical battery by waves, photolysis, and breakdown by microorganisms in the water (Andrady, 2011; Imhof et al., 2012). Larger plastic material is subsequently broken down into microplastics and nanoplastics (O’Brine and Thompson, 2010; Lambert et al., 2013; Gigault et al., 2016; Lambert and Wagner, 2016) which can be more concerning as a potential threat to organisms as microplastics and nanoplastics can pass through biological barriers (Mattsson et al., 2016), penetrate tissues, and accumulate in organisms (Von Moos et al., 2012). Current efforts focused on mitigating plastic pollution are aimed at cost-effective clean up and remediation technologies. For example, experiments on bioremediation of plastics has unveiled microbial biodegradation as a viable option to degrade polyethylene (PE), polystyrene (PS), and polyethylene terephthalate (PET) under specific conditions (Wu et al., 2017).

Macroplastics can be detrimental for aquatic mammals, impeding the digestive system or through entanglement, leading to early mortality. Compounding this issue, large plastic pieces are often degraded to smaller micro- and nanoplastic particles (MPs and NPs) by several mechanisms, including wave action, mechanical wear-and-tear, photo-oxidation, and microbial degradation (O’Brine and Thompson, 2010; Lambert et al., 2013; Cózar et al., 2014; Gigault et al., 2016; Lambert and Wagner, 2016). MPs and NPs can also enter the environment via industrial or residential effluents, or as components of cosmetics, industrial cleaners, or synthetic fibers among other sources (Oliveira et al., 2013; da Costa et al., 2016; Auta et al., 2017; Frias and Nash, 2019).

Marine and freshwater fish are often considered to be indicators of ecosystem health. Fish also comprise key components of aquatic the food chain and are a source of nutrition for both animals and humans. However, fish can also be highly sensitive to contaminants and are widely used as test species to conduct research on biological impacts of environmental contamination (Ahmad et al., 2012). Fish can be affected directly or indirectly by micro- and nanoplastic debris. Direct effects are often initiated at lower levels of biological organization (molecular level) while indirect effects can subsequently occur within the food chain and manifest as altered behavior of the individual (Adams et al., 1993). Several predatory fish are keystone species of aquatic food webs and as a result, accumulate significant amounts of plastic pollutants from the water and other organisms (Mansour and Sidky, 2002). An important point is that the accumulation pattern of plastic contaminants in fish can depend upon both the uptake and elimination rates of plastic debris, based on the physiology of the individual’s digestive tract (Hakanson, 1984).

Common carp (*Cyprinus carpio* L) is a widely cultured freshwater species worldwide (Xu et al., 2012). Common carp exhibit high tolerance to environmental stressors and is a model species for several research disciplines (Williams et al., 2008). While studies report adverse effects of plastic debris in carp (Hu et al., 2022; Liu et al., 2022), the effects of macro-, micro-, and nanoplastic-mediated inhibition of central nervous system (CNS) function, particularly in carp, remains unexplored. This is important because the brain maintains homeostasis of multiple physiological systems and contains high concentrations of polyunsaturated fatty acids. Such macromolecules are susceptible to free radical attacks following toxicant exposure (Şahin and Gümüşlü, 2004). Other molecules in the CNS susceptible for neurotoxicity include acetyl cholinesterase (AChE) and monoamine oxidase (MAO), each of which play an important roles for synaptic plasticity and neurotransmission (Liu et al., 2013). Nitric oxide (NO), synthesized by nitric oxide synthase (NOS), is also a significant endogenous mediator of biological activities in the CNS (Holmqvist and Ekström, 1997) and concentrations of NO are susceptible to change with chemical exposure.

Based on previous studies, we expect that our study will provide a new addition about the harmful effect of different sizes of plastic waste on the brain and sensory organs in fish. So in this study, juvenile common carp (*Cyprinus carpio*) were exposed to macro-, micro- or nanoplastics to determine their effect on biochemical and histopathological responses in the carp brain. Specifically, the objective of the study was to determine the biochemical mechanisms underlying macro-, micro-, and nanoplastic-induced neurotoxicity by assessing neurological parameters such as acetylcholinesterase (AChE), MAO and the content of nitric oxide (NO) activity, as well as conducted detailed histology on discrete brain regions.

## Material and methods

### Chemicals

Polyethylene macroplastics (MaPs; > 5 mm in size), microplastics (MPs; 5 mm > MPs > 100 nm in size), and nanoplastics (NPs; < 100 nm in size) were purchased as raw powders with irregular-shaped particles (Toxemerge Pty Ltd., Australia). Biochemical kits used in this study were acquired from Stanbio LDH (UVe Rate) and SGM Italia Co., USA (Hamed et al., 2022).

### Preparation of stock solutions

Parent stock solutions were first prepared at concentrations of 2 g/L for each size class in ultra-pure water (Milli-Q, 18.2 MΩ cm, 25°C). Stock solutions were stored in the dark at 4°C. To disaggregate the particles, parent stock solutions were sonicated using a SONICA-2200 E (20 kHz; 750 W) sonicator prior to each use. Dilutions were prepared for the 100 mg/L test solutions from the parent stocks immediately prior to the start of each experiment. Additional information on exposures can be found in recently published studies from our group (Hamed et al., 2019, 2022).

### Fish acclimation and exposure experiments

Common carp (*Cyprinus carpio* L.) juveniles, weight (4 ± 1 g) and length (5.5 ± 1 cm) were purchased from the Aquaponics unit at Al Azhar University (Assiut branch, Egypt). The physicochemical properties of the water were monitored in the tanks (100 cm × 70 cm × 50 cm), where acclimatization and exposure experiments were conducted. Mean value parameters of water for the experiment were 28.5°C, pH 7.4, 6.9 mg/L dissolved oxygen, 12:12 h (light:dark), and conductivity of 260.8 mM/cm. Exposure water was changed daily (40%) and re-dosed to purify it from fish waste.

Fish were divided into triplicate tanks over four treatments and there were 30 fish/treatment groups (i.e., 10 fish per tank). Experimental treatments included a control, and three treatments: 100 mg/L NPs, 100 mg/L MPs, and 100 mg/L MaPs (nominal concentration), this concentration was selected based on published studies (Hamed et al., 2019, 2022). Throughout the experiment, the concentration of plastics was maintained by adding the designated PE-containing solution with daily renewed water in each tank, as well as consideration that there is increasing concentrations of plastics in the marine environment (e.g., Green et al., 2017). The concentration was also chosen in order to compare to other studies (e.g., Choi et al., 2018). Exposures were conducted for 15 days (Katzenberger and Thorpe, 2015; Hamed et al., 2019). Following the exposure period, six individuals were randomly sampled from each experimental group. Fish were anesthetized on ice prior to sample collection (Wilson et al., 2009).

### Neurotoxicity markers

Brain samples were homogenized on ice with 10 volumes of cold Tris buffered saline (10 mM Tris–HCl, 0.1 mM EDTA-2Na, 10 mM sucrose, 0.8% NaCl, pH 7.4). The homogenate was centrifuged at 3,000 rpm at 4°C for 10 min. Acetylcholinesterase (AChE), MAO, and the content of nitric oxide (NO) were subsequently measured in the supernatant using commercial kits (Nanjing Jiancheng Bioengineering Institute, Nanjing, China). To normalize activity, protein concentrations were determined using bovine serum albumin as the standard in a Bradford protein assay (Bradford, 1976). The activity of AChE in brain homogenates was determined using an AChE kit based upon established methods (Ellman et al., 1961). The activity of 1 U of AChE was defined as the number of hydrolyzed micromoles of acetylthiocholine iodide per min per microgram of protein. AChE activity in the brain homogenates was expressed as units per milligram of protein (U/mg protein). The activity of MAO was determined by measuring the production of benzyl aldehyde from the reaction of MAO and substrate aniline hydrochloride based on established methods (Tabakoff and Alivisatos, 1972). One unit (U) of MAO activity was defined as the amount that increased the absorbance by 0.01 at 37°C (expressed as U/mg protein). NO content in brain homogenates was expressed as micromoles per milligram of hippocampus protein (μmol/mg protein) based on Stuehr et al. (1989). Each biological replicate (*n* = 6) was measured in triplicate.

### Histological and histochemistry analysis

Following the exposure period, the brain and eye of carp were rapidly dissected and immediately fixed in 10% neutral buffered formalin. Fixed organs were processed using a paraffin embedding technique and sections were produced at 5–7 μm thickness. Samples were stained using Harris’s hematoxylin and eosin stain solutions (H and E) and cresyl violet stain solutions. Histology was examined under a BX50F4 OLYMPUS microscope (Olympus optical Co., LTP. Japan).

### Statistical analysis

Biochemical data were first evaluated using the Shapiro-Wilk’s test for adherence to normality, and a Levene’s test was utilized to assess homogeneity of variance. A one-way analysis of variance (ANOVA) was used to test for differences among groups ffro the dependent variable, and a Tukey’s *post-hoc* test corrected for multiple comparisons. All statistical analyses were conducting using the SPSS package (SPSS, 1998) was used. An alpha level of 0.05 was used to accept or reject the null hypothesis. All data are presented as mean ± standard deviation (SD).

## Results

### Neurological parameters

The enzyme activity of acetylcholinesterase (AChE), MAO, and the content of nitric oxide (NO) were significantly decreased (*P* < 0.05) after exposure to 100 mg/L of either macroplastics, microplastics, and nanoplastic for 15 days compared to the control group (Table 1).

**TABLE 1:** Effect of (macro-, micro-, and nano) plastics exposure for 15 days on neurological biochemical endpoints of the Common carp (*Cyprinus carpio L*.).

Treatment	Control	Macroplastic	Microplastic	Nanoplastic
parameters				
Acetylcholinesterase (AChE) U/mg protein	5.2 ± 0.07 ^a^	4.1 ± 0.11 ^b^	3.6 ± 0.26 ^c^	3 ± 0.1 ^c^
Monoamine oxidase (MAO) U/mg protein	11.56 ± 0.15^a^	10.26 ± 0.20 ^b^	9.07 ± 0.15 ^c^	7.37 ± 0.15 ^d^
Nitric oxide (NO) mmol/mg protein	1.2 ± 0.065 ^a^	1.03 ± 0.026 ^a^	0.93 ± 0.04 ^b^	0.77 ± 0.015 ^c^

Data are represented as means ± SD. Values with different superscript letter in the same row for each parameter indicate groups are significantly different from controls (*P* < 0.05).

### Histological structure of the brain tissue

Several histopathological changes were noted in various layers of the optic tectum in common carp due to macroplastic, microplastic, and nanoplastic treatment. The severity and frequency of lesions in these layers were more pronounced in common carp treated with nanoplastics.

**FIGURE 1:**
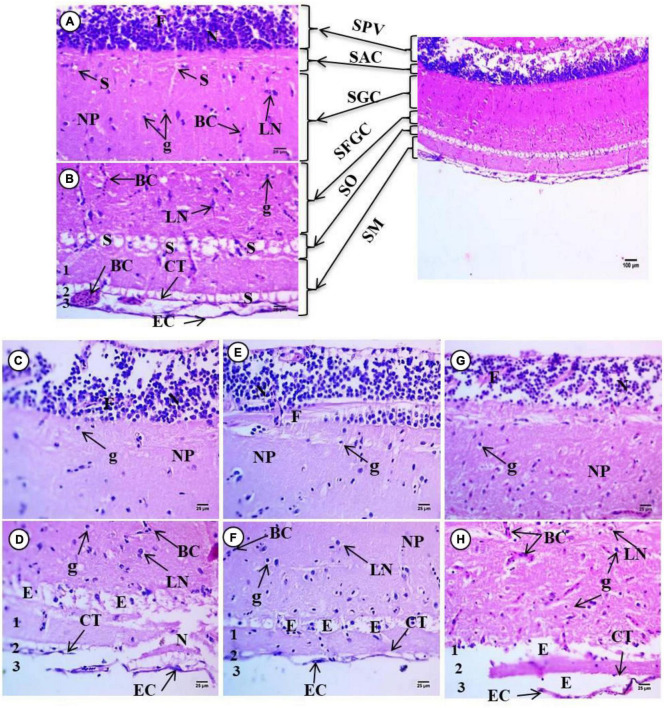
Representative sections of optic tectum of control and treated fish *Cyprinus carpio*; **(A,B)** sections of control fish brain showing Optic tectum of *Carp* intact zones, Stratum periventriculare (SPV) in the inner region, album centrale (SAC), followed by griseum centrale (SGC), fibrosum et grisium superficiale (SFGS), opticum (SO), and marginale (SM). **(C,D)** Exposure to 100 mg/L of macroplastic for 15 days showing necrosis (N) and fibrosis **(F)** in the granular cells of the SPV zone, spongiosis/edema (E) of granular cells of the SFGC zone and degenerated connective tissues (CT) and degenerated outer squamous cells (EC); **(E,F)** exposure to 100 mg/L of microplastic for 15 days showing detachment and high degeneration of large neuronal cells (LN) and glial ganglion cells (g) of the SFGS, edeama (E) of granular cells of the OS zone and degenerated connective tissues (CT) and outer squamous cells (EC). **(G,H)** Exposure to 100 mg/L of nanoplastic for 15 days showing necrosis (N) and fibrosis (F) in SPV zone, increase in blood capillaries (BC), detachment and necrosis in large neurons (LN) and ganglion cells (g) in the SFGS zone, edema in SO zone and degenerated connective tissues (CT) and outer squamous cells (EC) **(H&E × 400)**.

The optic tectum of C. carp is a bilobed structure located in the dorsal part of the mesencephalon. This region of the CNS acts primary as the visual center; six different layers are present, each containing various shapes and sizes of neurons. In untreated fish, moving from the ependyma to the outer surface, the following structures are observed: The stratum periventriculare (SPV) is in the inner region characterized by deeply stained nuclei connected by fibers and glial cells, and the second layer album centrale (SAC) contains acidophilic staining neuropile (NP) with small unstained spaces or spongosis (S) and glial cells. The third layer, griseum centrale (SGC), consists of acidophilic neuropile with blood capillaries (BC) localized beside a large unstained area. In this region, mononuclear nuclei of glial cells (g) and a few large neurons with vesicular nuclei (LN) can be observed in this unstained space. The fourth layer, fibrosum grisium superficiale (SFGS), contains heterogeneous acidophilic neuropile with many small irregular unstained space(s), several large neurons with deeply stained nuclei (LN), and small glial cells with nuclei and few blood capillaries. The fifth layer, stratum opticum (SO), appears as unstained regions containing neuropile as a network, contains unstained space (S) rich with blood capillaries. The final outer layer, stratum marginale (SM), consists of several smaller layers. There is the internal layer which contains acidophilic neuropile with a large region of spongiosis (S) with blood capillaries and glial cells with small nuclei, an unstained region, and an external layer that consists of connective tissue, epithelial cells (EC), and blood capillaries (BC) (Figures 1A,B).

The tissue showed marked structural differences in the treated fish. There was necrosis and detachment of granular cells in the SPV zone, spongiosis of granular cells of SFGC zone, necrosis in the outer layer, degeneration of connective tissue, edema, elevated and degenerated outer squamous cells following exposure to 100 mg/L macroplastic (Figures 1C,D). These changes were most pronounced in individuals exposed to 100 mg/L microplastics (Figures 1E,F) and included detachment and necrosis in the granular cells of SPV zone, necrosis in mononuclear cells, as well as detachment and high degeneration of neuronal cells of the SO and SM lining. Pathology was most pronounced in fish treated with 100 mg/L nanoparticles (Figures 1G,H), and observations included spongiosis of granular cells of SPV zone and vacuolization in the granular cells of SAC layer due to degeneration. There was also increased numbers of blood capillaries in the SFGC zone and reduction of the SO and SM layers due to the degeneration of neuronal cells.

### Histochemistry of brain tissue (optic tectum)

**FIGURE 2:**
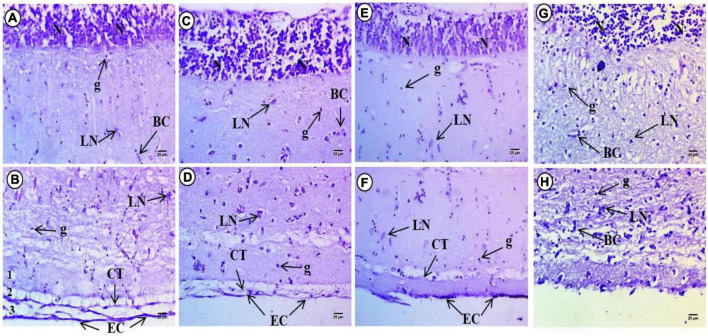
Sections of control and treated fish brain tissue. **(A,B)** Sections of control fish brain showing high intensity in internal layer of small neuronal cells (N), large neurons (LN) with faint basophilic Nissl bodies. Deeply stained neurons beside glial cells (g) and deeply stained violet color in the covering connective tissues (CT) and epithelial cells (EC). **(C,D)** Exposure to 100 mg/L of macroplastic for 15 days showing intensity of violet color in small neuronal cells (N), dispersed different shapes of large neurons (LN) and deeper staining in the covering connective tissues (CT) and epithelial cells (EC). **(E,F)** Exposure to 100 mg/L of microplastic for 15 days showing slight reduction in color intensity of internal layer small neurons (N), dispersed degenerated large neurons (LN) and less staining in the covering connective tissues (CT) and epithelial cells (EC). **(G,H)** Exposure to 100 mg/L of nanoplastic for 15 days showing a slight increment in color intensity of internal layer small neurons (N). Dispersed degenerated large neurons (LN) and deep staining in the covering connective tissues (CT) and epithelial cells (EC); **(Cresyl violet Stain × 400)**.

Sections of the optic tectum of control *Cyprinus carpio* stained by Cresyl violet showed a high intensity of violet color in the internal layer containing small neuronal cells based on staining of Nissl bodies. The second to fourth layers contains few large neurons with faint basophilic Nissl bodies. The fifth layer contained deeply stained neurons as well as glial cells, while the last layer showed deeply stained violet color in the covering connective tissues and epithelial cells (Figures 2A,B).

Examination of brain sections with Cresyl violet after 15 days macroplastic exposure showed strong intensity of violet color and small neuronal cells in the internal layer which appeared less compacted and filled with ribonucleic acids. The second to fourth layers contained dispersed large neurons of different shapes with deeply stained basophilic Nissl granules perinuclei. The fifth layer contained deeply stained neurons at the border between the fifth and six layers while the last later showed deeper staining relative to the control groups (Figures 2C,D). Examination of brain sections with Cresyl violet after 15 days of microplastics showed a slight reduction in color intensity of the internal layer with small neurons, whereas in the second to fourth layers, there were dispersed degenerated large neurons with deeply stained basophilic ribonucleic acid perinuclei. The fifth layer contained deeply stained aggregated neurons, while the last layer showed reduced staining overall compared to the control groups (Figures 2E,F).

Examination of brain sections stained with Cresyl violet after 15 days exposure to nanoparticles showed a slight increment in color intensity of the internal layer of small neurons. The second to fourth layers contained dispersed and degenerated large neurons with deeply stained basophilic ribonucleic acid perinuclei. The fifth layer also contains deeply stained aggregated neurons and showed reduced staining relative to the tectum of control fish (Figures 2G,H).

### Histological structure of the eye

**FIGURE 3:**
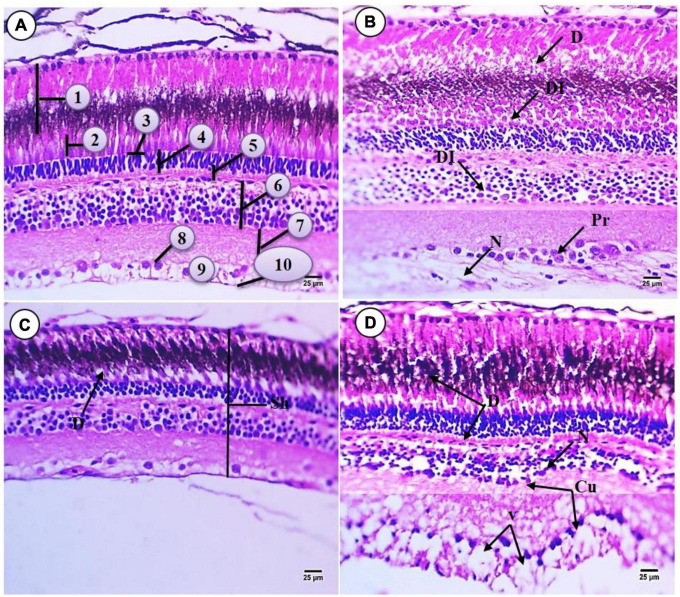
Sections of retina of control and treated fish *Cyprinus carpio*; **(A)** sections of control fish retina showing retinal layer: (1) RPE mostly contains melanin, (2) photoreceptor layer (PL) (rod and cone processes), (3) outer liming membrane (OLM), (4) outer nuclear layer (ONL) consisting of the nuclei of the photoreceptors, (5) OPL, (6) INL, (7) inner plexiform layer (IPL), (8) GL, (9) NFL, (10) internal liming membrane (ILM); **(B)** exposure to 100 mg/L of macroplastic for 15 days showing necrosis (N), degeneration (D), disintegrated (DI) and proliferation (Pr); **(C)** exposure to 100 mg/L of microplastic for 15 days showing shrunken (Sh) in retinal layer and degeneration (D); **(D)** exposure to 100 mg/L of nanoplastic for 15 days showing necrosis (N), degeneration (D), vacuolation (V) and curved in inner layer (Cu) **(H&E × 400)**.

Based on H&E staining, the histological micrograph of control common carp eye was composed of three layers comprising an inner, middle, and external layer. The inner layer of the eyes is the retina which is composed of 10 layers arranged in the following sequence (from inner to outer) (Figure 3A): The Retinal Pigment Epithelium (RPE) is the outer layer of the retina consisting of non-neuronal cells of simple cuboidal epithelium with rounded, large and centrally located nuclei containing melanin pigment. This layer was close to the choroid layer. The Photoreceptor layer (PL) contains the rods and cones. The outer segment of these cells contains packed photoreceptor components that stain acidophilic while the middle region contains deeply stained basophilic materials. Rod cells are long and ellipsoid in shape while the cone cells are conical and a bulbous ellipsoid. The function of rod cells is to sense light and dark conditions while the function of the cone cells is to generate information about the color spectrum. Cell bodies of these photoreceptors are arranged to comprise the outer nuclear layer. The junctional complex between photoreceptors and glia cells are arranged as a thin line creating the outer Limiting Membrane (OLM) which is located above this line with acidophilic cytoplasm and constitutes the basal part of the photoreceptor cells. The Outer Plexiform Layer (OPL) includes the synapses between photoreceptor cell processes and bipolar, horizontal, and amacrine cells of the inner nuclear layer (IPL). The IPL includes the synapses between bipolar cells, axons, and ganglion cells. The Ganglion Cell Layer (GL) contains large cell bodies comprised of a narrow layer of granular and spherical cells surrounded by a fine network of connective tissue. The Nerve Fiber Layer (NFL) is a layer that includes axons of ganglion cell. These cells project and merge into the optic disc to form the optic nerve. Lastly the Internal Limiting Membrane (ILM) forms gelatin like connective tissues.

Exposure to macroplastics resulted in adverse impacts on the retina morphology including the following observations: The outer pigment epithelium appeared irregular, and there were damaged cells containing unstained cytoplasm with deformed shape and shrunken nuclei. Degeneration was also observed in retinal epithelial cells which contain acidophilic cytoplasm, deeply stained basophilic fine granules, and necrosis. The photoreceptor layer lost its regular distribution and contained deformed rods and cones. There was the disappearance of OLM and the external limiting membrane. Also observed was that the nuclear layer lost their morphology, with evidence for hydropic degeneration (edematous) with deeply stained nuclei (pyknotic). There was also a slight widening of the OPL and irregular distribution of their cells. In addition, the nuclear layer cells were vacuolated with shrunken and different shapes of nuclei, and there were large and small pyknotic cells in unstained cytoplasm with a thickening region of acidophilic staining. Lastly, the inner plexiform layer did not appear affected but the GL was increase or aggregated and was surrounded by unstained space. Compared to the controls, there was also increased width of NFL and irregular ILM (Figure 3B).

All retinal layers were deformed and shrunken with exposure to 100 mg/L microplastic. The pigment in epithelial cells was more or less similar to the flat epithelium and this deformation was observed in the retinal epithelium cells which contained deeply stained heterogonous cytoplasm with vacuoles (indicated by the blue arrow) at their apex. Also observed was the vacuolization of the layer of photoreceptor cells (indicated by the thin dark arrow) which contains rods and cones. There was faint staining of the OLM. The IPL decreased in height and their nuclei changed in shape to rounded or ovoid nuclei which were deeply stained basophilic (pyknosed) and surrounded by unstained areas as compared with controls. The OPL was degenerated and contained patches of spogiosis with faint acidophilic cytoplasm. The outer nuclear layer contained different types of neuronal cells surrounded by vacuoles. Necrosis was observed in the OPL matrix and appeared acidophilic in staining. Degeneration was also observed in the GL while necrosis was observed in the fiber layer which was decreased in width and contained border deeply stained indicating the inner limiting membrane (Figure 3C).

Fish exposed to 100 mg/L nanoparticles for 15 days showed severe RPE damage. The pigment and photoreceptor shape and height were similar to the controls but also contained a layer of degeneration and disintegrated tissue at their basal region. Severe damage and the disappearance of the OLM were observed and severe damage was observed in the outer nuclear layer, which was decreased in height and contained very small cells deeply stained (pyknosis). The OPL slightly decreased in area when compared with group (B), and the IPL and inner plexiform layer were irregular and contained signs of degeneration and necrotic areas. Cells were also shrunken with edema (E) while the matrix of the IPL was increased in width and appeared like meshwork. Irregularity of the ganglion cell and NFL was observed, and ganglion cells were deeply stained basophilic and surrounded by large vacuoles, degeneration, and necrosis. There was also increased width of GVL and NFL compared to all other groups. There was the disappearance or degeneration of ILM (Figure 3D).

### Histochemistry (cresyl violet for Nissl bodies or RNA) in retinal layer

**FIGURE 4:**
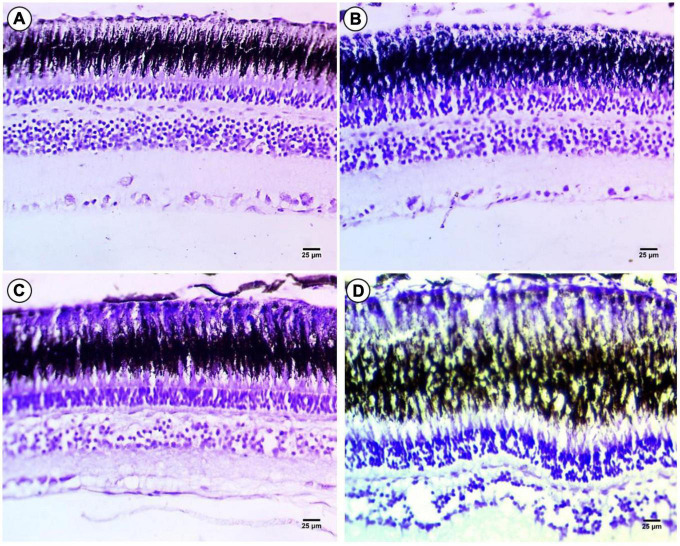
Sections of control and treated fish retina. **(A)** Sections of control fish retina showing positive staining in all layers of the retina of fish. **(B)** Exposure to 100 mg/L of macroplastic for 15 days showing slight positive staining in the four layers of the retina. **(C)** Exposure to 100 mg/L of microplastic for 15 days showing negative staining in the four layers of the retina. **(D)** Exposure to 100 mg/L of nanoplastic for 15 days showed negative staining in all layers of the retina **(cresyl violet stain × 400).**

Cresyl violet staining was employed for Nissl bodies or (RNA) and a violet color indicates positive staining in all layers of the fish retina compared to the control group (Figure 4A). In fish treated with 100 mg/L macroplastics, there was an intensive color observed in the photoreceptors, but in the outer and IPLs, there was a depletion of color stain, while in the outer and inner plexiform layers, there was depletion in staining especially in the GLs (Figure 4B). In microplastic-treated fish, it was observed that the photoreceptor layer and outer nuclear layer showed intense staining with Cresyl violet due to increasing amounts of RNA, while the IPL, outer and inner plexiform, and GLs showed significant depletion in color stain (Figure 4C). Lastly nanoplastic treated fish showed a significant decrease in staining in all layers with localization of coloration at the top of the RPE and outer nuclear layers (Figure 4D).

## Discussion

Freshwater fishes are affected by plastic materials derived from industrial activity, agricultural applications, and daily consumer activities; this leads to water pollution with toxic macro-, micro- and nanoplastics and this can negatively affect both aquatic organisms and humans (Hamed et al., 2019, 2020, 2021a,2021b,^2022^; Sayed et al., 2021a,b).

Experimental studies demonstrate detoxifying enzymes have critical roles in biological response to toxicants (Hao et al., 2008; Xing et al., 2013). For example, acetylcholinesterase or AChE, is a target of many environmental contaminants and is considered a useful biomarker for neurotoxicity (Pretto et al., 2010; Pérez and Rodríguez del Bosque, 2013). Our data showed that AChE activity was significantly decreased with macro, micro, and nanoplastic exposure in the brain of common carp. AChE controls synaptic transmission at cholinergic synapses by regulating levels of acetylcholine in the synaptic cleft (Basha et al., 2012). The inhibition of AChE activity by different size of plastics (Nano, Micro and Macro) may lead to oxidative stress in brain tissue and result in the accumulation of ACh at cholinergic synapses. This has consequences in the form of overstimulation of muscarinic and nicotinic receptors, decreases the cellular metabolism, and loss of cell membrane integrity which can disturb metabolic and nervous activities (Chen et al., 2017; Jung et al., 2020; Pedersen et al., 2020; Prüst et al., 2020; Sökmen et al., 2020). When AChE activity is decreased, acetylcholine accumulates within the synapse, altering neurotransmitter signaling. This can have downstream implications for the behavior of fish, impacting swimming and feeding (Glusczak et al., 2006). AChE inhibition is also correlated with cell damage and reduced AChE expression in the nervous system (Xing et al., 2013). Heavy metal exposure also causes significant impacts on AChE enzyme activity; for example AChE decreases in activity after cadmium exposure in silver catfish brain and muscle (Pretto et al., 2010). Taken together, our data suggest that MPs and NPs exposures induce significant neurotoxicity in carp brain.

Our data also revealed that exposure to plastic debris can lead to a decrease in MAO activity. MAO is involved in the metabolism of neurotransmitters such as dopamine, norepinephrine, epinephrine, and serotonin (Devi et al., 2005; Prüst et al., 2020) Dysfunctions in both AChE and MAO are associated with several neuropsychiatric disorders (Liu et al., 2013) and the inhibition of AChE and MAO activities with different sizes of plastics (Nano, Micro and Macro) may lead to oxidative stress and higher inhibition of their function (Reddy et al., 2007; Chen et al., 2017; Barboza et al., 2018; Iheanacho and Odo, 2020). In this study, the MAO level decreased significantly in exposed fish compared with the control. In contrast, Basu et al. (2007) stated that a negative correlation was calculated between the concentrations of brain Hg (i.e., total Hg and MeHg) and the activities of MAO in the cerebral cortex of North American river otters. Also, Li et al. (2015) found that TBT decreased NO production in the brains of exposed juvenile common carp (Cyprinus carpio). Mukherjee et al. (2019) observed a statistically significant difference in the mean MAO level between all the different treatments of pH and type of carp. Li and Li (2021) found MAO activities were significantly decreased compared to control in brain tissues of zebrafish exposed to TBT concentrations at 100 and 300 ng/L.

Nitric oxide (NO) regulates cell signaling and neurotransmission in several cells (Nava-Ruíz et al., 2010). Disruptions in NO production may therefore be a causal factor in the development of neurotoxicity (Kim et al., 2011). On the other hand, higher concentrations of NO can be toxic to cells, interacting with the superoxide anion to produce the highly toxic peroxynitrite anion (Traystman et al., 1991). In carp, we found that different sizes of plastic (Nano, Micro and Macro) decreased brain NO levels in exposed fish. Other studies observed that different size of plastic (Nano, Micro and Macro) markedly increased ROS levels in fish brains (Chen et al., 2017; Jung et al., 2020; Sökmen et al., 2020). In the CNS, the production of ROS can reduce NO levels as NO reacts rapidly with O2- to produce the peroxynitrite anion (ONOO-), This reactive molecule can then protonate at a given pH to form peroxynitrous acid (ONOOH) (Liu et al., 2013). Both ONOO- and ONOOH cause nitrosative stress, leading to nitrosylation reactions that alter the structure of proteins and impair their function (Nava-Ruíz et al., 2010). Such impacts of MPs and NPs are similar to those reported with heavy metal and other plastic studies exposures in fish (Guilhermino et al., 2000; Pretto et al., 2010; Barboza et al., 2018; Hamed et al., 2020, 2022).

The CNS regulates all aspects of physiology, and its morphology is susceptible to damage from environmental pollutants. Anatomical and histological microstructures of the brain differ across species of fish, but the role of specific brain regions is relatively conserved across species (Abdelnaeim Hussein and Cao, 2018). With MPs and NPs treatment, it was observed that carp treated at a concentration of 100 mg/L of macroplastics showed several histopathological changes, including necrosis, spongiosis, degeneration, and edema. These findings agree with other studies (Hamed et al., 2021b) that reported several changes in the brain based on histopathology, including tissue degeneration, vacuolar degeneration, edema, necrosis, and hemorrhage in tilapia after exposure to microplastic. Similar effects were reported in goldfish (*Carassius auratus*) exposed to virgin microplastics and zebrafish (*Danio rerio*) larvae exposed to pristine low-density polyethylene fragments (Karami et al., 2017; Romano et al., 2020).

In the optic tectum, exposure to 100 mg/L of microplastic for 15 days resulted in pathology that was more severe. This included detachment and necrosis in the granular cells of the SPV zone, mononuclear cell necrosis, detachment and notable degeneration of neuronal cells contained within the SO and SM lining. These changes parallel those observed in Africa catfish that were exposed to the herbicide glyphosate (Erhunmwunse et al., 2014) and C*yprinus carpio* exposed to quinalphos (Chamarthi et al., 2014).

Exposure to a concentration of 100 mg/L of nanoplastic for 15 days resulted in spongiosis of granular cells of SPV zone and vacuolization in the granular cells of SAC layer due to degeneration. There was also an increase in the number of blood capillaries in the SFGC zone and a loss of SO and SM lining due to detachment and high degeneration of neuronal cells. In another study, brain tissues of the spotted grouper *Epinephelus coioides* paralleled histopathology observed here following exposure to methylmercury, and noted was hyperemia, hemorrhage, karyolysis, tissue necrosis, hyper-chromatin, vacuolation, endothelium hypertrophy, hydropic degeneration, and ectopic granular accumulation (Savari et al., 2020). Results of the current study and that in spotted grouper suggest that heavy substances may have similar effect on the brain.

In addition, several authors have also reported histopathological alterations in the CNS of fishes following exposure to microplastic and various chemical substances (Das and Mukherjee, 2000; Ayoola and Ajani, 2008; Pugazhvendan et al., 2009; Karami et al., 2017; Lakshmaiah, 2017; Romano et al., 2020).

In the present study, the histology of the retina of carp revealed varying degrees of damage that corresponded to macro, micro and nanoplastic in all groups. Alterations included degeneration, increases in the number and size of some layers, disintegration of tissue, necrotic areas, and the presence of large vacuoles. These findings are in agreement with Francis and Nagarajan (2013) who observed that goldfish did not move their eyes in polluted waters. In addition, Hofer and Gatumu (1994) reported that, in *Oncorhynchus mykiss*, there was a range of optic pathology from necrosis of single retinal neurons to complete destruction of the retina when fish were exposed to sub-lethal concentrations of nitrite (Hofer and Gatumu, 1994). Other studies report that exposure to environmentally relevant levels of PVC-MP can cause oxidative damage in the brain and liver, adverse morphological/anatomical changes to the brain, intestine and liver and altered gene expression in goldfish (Romano et al., 2020). Our observations of degenerated retina are also in agreement with other studies and toxicological agents that include polychlorinated bisphenols Lavakumar (2003), municipal effluent exposures with *Clarias batrachus* (Nagarajan and Bhuvaneswari, 2009), and untreated and treated sago effluent exposures with *Cirrhinus mrigala* and *Clarias batrachus* (Nagarajan and Suresh, 2005; Ramesh and Nagarajan, 2013) among other studies.

### Conclusion

Here we report on the effects of varying sizes of polyethylene plastic particles in the common carp using a neurotoxic biomarker approach to evaluate their acute toxicity. Both neurotoxic biomarker and histological changes were observed in carp following exposure to different sizes (NPs, MPs, and MaPs) of PE plastics. Responses at the biochemical and histopathological level are consistent with neurotoxic effects observed in fish treated with other types of environmental toxicants like metals and metallic nanoparticles. We noted that the biomarkers measured were altered in the sole presence of PE particles and that the size of the particle is important for the biological response levels to NPs exposure. The effect of size may be related to the ability to smaller plastics to enter cells and tissues, inducing molecular damage due to their physical presence in the tissue, as well as the chemicals that comprise the plastic particles themselves. This study addresses a knowledge gap concerning the potential risks of micro- and especially nanoplastics for neurotoxicity in aquatic organisms. Through this study, we suggest conducting scientific experiments aimed to reducing the biological damage caused by plastic waste to aquatic organisms.

## Data availability statement

The original contributions presented in this study are included in the article/supplementary material, further inquiries can be directed to the corresponding author.

## Ethics statement

The animal study was reviewed and approved by the Assiut University Committee.

## Author contributions

MH and AE-DS: conceptualization, methodology, visualization, and investigation. MH, AE-DS, and MN: data curation and writing – original draft preparation. MH, CM, MN, J-SL, and AE-DS: final draft writing – reviewing and editing. All authors contributed to the article and approved the submitted version.
